# Phenotypic and transcriptomic responses of two *Nilaparvata lugens* populations to the Mudgo rice containing *Bph1*

**DOI:** 10.1038/s41598-019-50632-z

**Published:** 2019-10-01

**Authors:** Pin-Jun Wan, Ruo-Nan Zhou, Satyabrata Nanda, Jia-Chun He, San-Yue Yuan, Wei-Xia Wang, Feng-Xiang Lai, Qiang Fu

**Affiliations:** State Key Laboratory of Rice Biology, China National Rice Research Institute, Hangzhou, 310006 China

**Keywords:** Zoology, Animal physiology

## Abstract

The *Bph1* gene was the first reported brown planthopper (BPH, *Nilaparvata lugens*) resistance gene in Mudgo rice and was widely used as a commercial cultivar for controlling BPH infestations. However, rapid adaptations of BPH on the Mudgo rice resulted in its resistance breakdown and the emergence of virulent BPH populations. Thus, specific BPH populations and rice varieties can serve as good model systems for studying the roles of different bio-compounds and proteins in the insect-plant interactions. Although our understandings have been improved on the complexity of BPH and rice interactions, the underlying molecular mechanisms remain largely unknown. Here we analyzed the feeding performances and the transcriptomic responses of two BPH populations (Mugdo-BPH and TN1-BPH) during compatible (Mudog-BPH feeding on Mudgo rice) and incompatible (TN1-BPH feeding on Mudgo rice) interactions. The electrical penetration graph (EPG) results indicated that the BPH feeding and performances during the incompatible interaction are significantly affected in terms of decreased honeydew, loss of weight, decreased phloem sap ingestion (N4 waveform), but increased non-penetration (NP waveform) phase. Abundance of glucose and trehalose was reduced in BPH during the incompatible interaction. Transcriptomic surveys of insects in both interactions revealed that genes involved in cuticle formation, detoxification, metabolite transport, digestion, RNA processing, lipid or fatty acid metabolism, and proteolysis were significantly down-regulated during the incompatible interaction, whereas genes involved in insulin signaling were significantly upregulated. Knockdown of four genes, including the sugar transporter *NlST45*, the serine and arginine-rich protein *NlSRp54*, the cytochrome P450 gene *NlCYP6AY1*, and the cuticle protein *NlCPR70* through RNA-interference revealed thess genes are important for BPH survival. Overall, the results of this study will be helpful for the future researches on BPH virulence shifts.

## Introduction

*Nilaparvata lugens* Stål, commonly known as the brown planthopper (BPH) is a hemipteran pest of rice causing severe crop losses throughout Asia. Being a typical monophagous phloem feeder, it not only directly damages the rice plants, often leading to plant wilting and subsequent death known as ‘hopper burn’, but also act as a carrier for the grassy stunt and ragged stunt viruses^1^. Although countering the BPH infestation by adopting the integrated pest management strategies are intended, the use of pesticides against BPH remains the most common practice at present^2^. However, the widespread insecticide utilization in rice production inevitably causes environmental risks and the resurgence of planthopper^3,4^. On the other hand, many rice varieties carrying specific genetic resistance genes (*Bph/bph*) have been used to control BPH damage. To date, 34 *Bph* genes have been identified in rice and its wild relatives^5,6^. Out of these, *Bph1* was the first reported gene in the *indica* cultivar Mudgo in 1969^7^, and has been mapped between the odQNP and odVMN markers on chromosome 12 at distances of 7.5 cM and 8.4cM^8–10^. Since then, more than twenty cultivars harboring the *Bph1* gene were developed and used as the commercial cultivar for controlling BPH infestations^9,11^. Initially, the Mudgo rice exhibited resistance to BPH as evidenced from the slow growth rate, small body size, and low fecundity rate of BPH^12^. However, BPH resistance in resistant rice cultivars has broken down from the emergence of the different virulent BPH populations^13,14^. Although, the underlying mechanisms for this shift in virulence is still unclear, some major causes of it might be the emergence of *virulence* genes or effectors in BPH^15,16^, assistance from the endosymbionts^17^, dietary changes due to change in host rice types^18^, and selective changes in BPH metabolisms^19^.

Resistant rice varieties have been reported to have significant effects on the overall BPH fitness, including feeding periods, survival, metamorphosis, and fecundity^20,21^. For instance, during the compatible rice-BPH interactions, probing and pathway (N_1_ + N_2_ + N_3_) phases have been reported to be much shorter as compared to that of during the incompatible interactions^22^. Conversely, the phloem feeding phases (N4) are significantly longer during the incompatible rice-BPH interactions than the compatible ones^22^. Similarly, production of honeydew, another indicator of BPH fitness on rice, was reported to be significantly higher during the compatible interaction than that of the incompatible one^22^. Additionally, phloem sap feedings during the BPH-rice interactions can affect the BPH metabolism and physiology. A previous report showed that during the Mudgo-BPH feedings on Mudgo rice, the induced gene expressions related to nucleotide/amino-sugars and carbohydrate metabolisms were observed^23^. Further, replacing the TN1 rice (susceptible to BPH) with the resistant rice variety B5 (carrying *Bph14* and *Bph15*) during the BPH feedings resulted in the upregulated expression of triacylglycerol lipase, ribonucleoside hydrolase genes, and genes involved in the trehalose synthesis^24^. Moreover, TN1-BPH nymphs feeding on a near-isogenic line carrying *Bph15* gene have upregulated expression of *trehalose 6-phosphate synthase* gene, encoding the enzyme that converts trehalose from glucose, and elevated levels of trehalose as compared to the nymphs feeding on a susceptible rice^21^. On the contrary, nymphs feeding on the susceptible rice contained higher amount of glucose than those feeding on a resistant variety^21^. Thus, these observations suggest that the nature of rice-BPH interactions (compatible/incompatible) have significant effects on BPH metabolisms, in particular on the major sugars like glucose and trehalose.

The BPH infestations on specific rice varieties affect the reprogramming of numerous genes in BPH resulting in a change in the mRNA pool. Several studies have been conducted to identify the key regulating genes or effectors by analyzing the insect transcriptomes during their infestation periods, including *Myzus persicae*^25^, *Bemisia tabaci*^26^, *Empoasca fabae*^27^, and *N. lugens*^23,24^. The initial records of the BPH transcripts came in the picture, when Noda *et al*. reported exceeding of 37,000 expressed sequence tags (ESTs) from various tissues of *N. lugens*^28^. Subsequently, comparative transcriptome analysis of the salivary gland of two separate BPH populations having different virulence levels were studied^23^. Furthermore, about 67 genes encoding putative secretive proteins in the salivary glands of BPH were found to show differential expression patterns in between the two BPH populations. Similarly, Wang *et al*. reported the differential transcriptomes in the BPH salivary glands after feeding on a resistant and a susceptible rice variety^24^. Besides, the BPH feeding on a susceptible rice was reported to have more number of proteins related to digestive functions as compared to the BPH feeding on a resistant rice^29^. Although, much attention has been given to analyze the differential transcriptomes of the BPH salivary glands, only a few studies have been carried out to explore the whole transcriptome profiles. As feeding on different rice varieties have significant effects on the BPH overall fitness, including growth, survival, and fecundity, examining the whole body transcriptomes of different BPH populations will offer a great deal of understandings on rice-BPH interactions.

In this study, two different BPH populations i.e. TN1 population (TN1-BPH, reared and maintained on TN1 rice) and Mudgo population (Mudgo-BPH, reared and maintained on Mudgo) rice have been fed on Mudgo rice. The feeding behaviors of both BPH populations have been recorded by using the electrical penetration graph (EPG) technique. Further, the amounts of honeydew secreted by the two BPH populations were observed. In addition, biochemical analysis of two major sugar pathways, including glucose and trehalose were conducted for both of the BPH populations. Lastly, whole body transcriptomic profiles of the TN1-BPH and Mudgo-BPH were sequenced and studied upon feeding on the resistance Mudgo rice. Moreover, the results of this study will add further insights to the understanding of rice-BPH interactions and differential BPH virulence.

## Result

### BPH performances on the Mudgo rice variety

**Figure 1:**
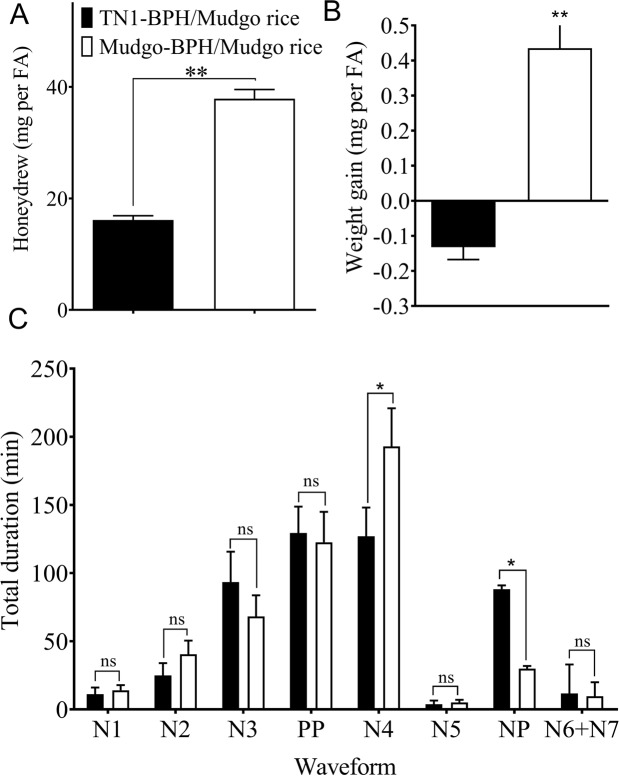
The growth rates (**A**), honeydew weight (**B**) and feeding behavior (**C**) of TN1-BPH and Mudgo-BPH on Mudgo rice. The newly emerged (0 h) BPH and those feeding on Mudgo rice for 48 hours were collected to determine the fresh body weight, the honeydew, and to record the feedings for 6 h on a CR-8 direct current (DC) EPG amplifier. Asterisks indicate a statistically significant difference between TN1-BPH and Mudgo-BPH (*P* < 0.05, Student’s *t*‐test).

In order to reveal the performance of BPH populations on the *Bph1*-harboring Mudgo rice, the growth rates and feeding behaviors of TN1-BPH and Mudgo-BPH were analyzed. The TN1-BPH infested on the Mudgo rice excreted significantly less honeydew (*P* < 0.001) (Fig. 1A) than the Mudgo-BPH, as well as having negative weight gain values (*P* < 0.001) (Fig. 1B). As analyzed via EPG (Fig. 1C), three (“pathway” phase, PP; xylem ingestion phase, N5; the unclear phase, N6 for derailed stylet mechanics and N7 for cell penetration) out of the five main phases of BPH feeding showed no significant differences between TN1-BPH and Mudgo-BPH. However, the Mudgo-BPH individuals spent significantly more time in continuously ingesting phloem sap (N4 phase), but less time in the non-penetration (NP) phase as compared with the TN1-BPH. These results indicated that infestation of the avirulent TN1-BPH on the *Bph1*-harboring Mudgo rice significantly reduced the BPH feeding and performance.

### Trehalose, glucose and glycogen contents in the BPH infested on Mudgo rice

**Figure 2:**
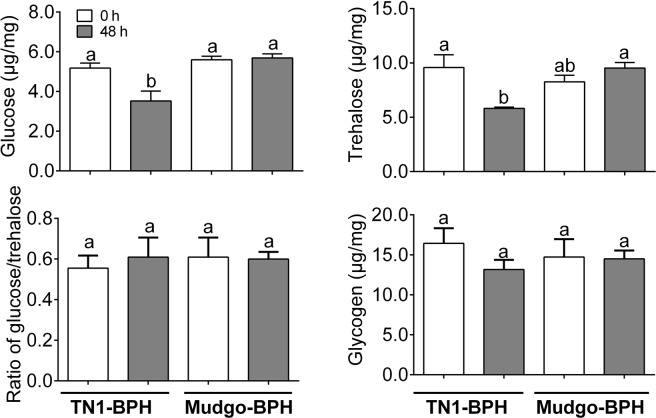
The glucose (**A**) and trehalose (**B**) contents, ratio of glucose/trehalose (**C**), and glycogen (**D**) content in the TN1-BPH or Mudgo-BPH female adults. The newly emerged (0 h) BPH and those feeding on Mudgo rice for 48 hours were collected. The contents of trehalose, glucose and glycogen are measured. Data are denoted as mean ± SE and analyzed by two-way ANOVA followed by Tukey’s HSD post-hoc test. Different letters indicate significant differences at *P* value < 0.05.

**Figure 3:**
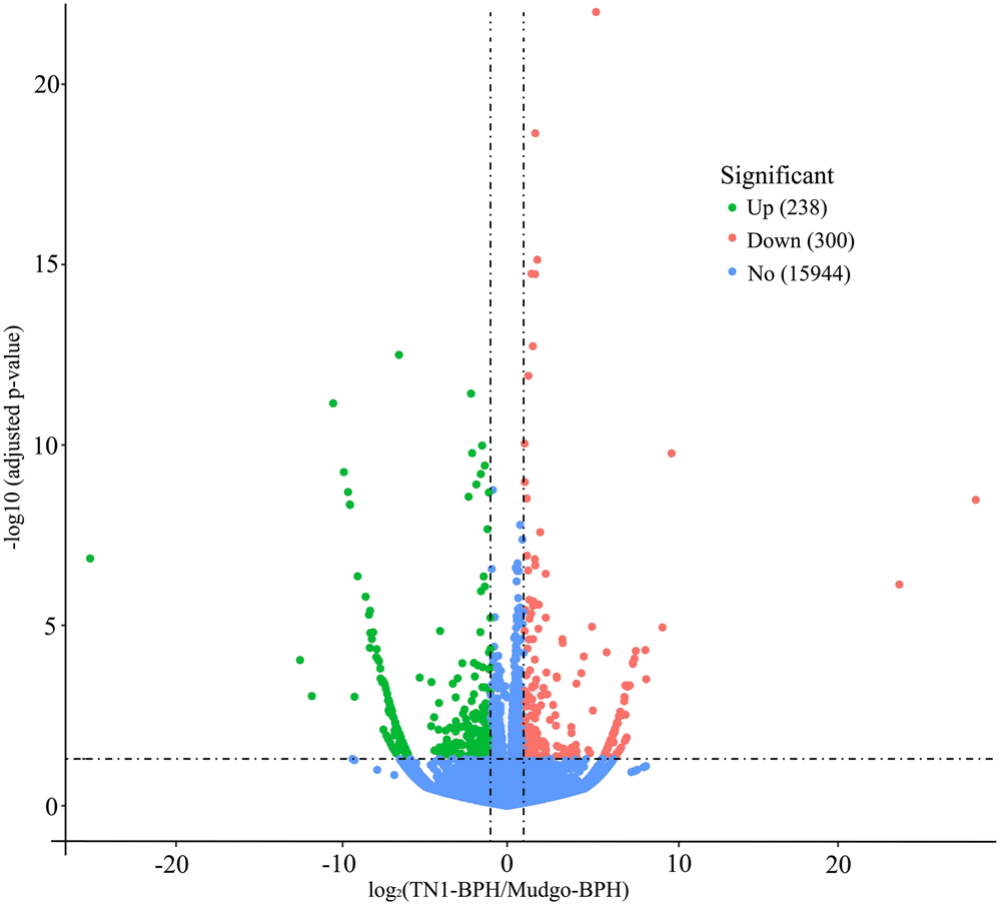
Differentially expressed genes in TN1-BPH and Mudgo-BPH feeding on Mudgo rice for 48 h. The significantly upregulated, and downregulated genes BPH were shown in red and green, respectively (adjusted *p*-value < 0.05). No differential expression between the two group of BPH genes was shown in blue (adjusted *p*-value > 0.05). The number of genes in each group was parenthesized.

To evaluate the effects of the rice-BPH interactions on the glucose metabolism, the trehalose, glucose and glycogen contents were determined in the TN1-BPH and Mudgo-BPH fed on the Mudgo rice. BPH feedings on the Mudgo rice for 48 h resulted in the significant lower glucose and trehalose contents the newly emerged female adults of TN1-BPH, whereas for the Mudgo-BPHs, no significant differences were observed (Fig. 2A,B). Moreover, the ratio of glucose/trehalose in the examined samples showed no significant difference (Fig. 2C). In BPH, glucose can be used to biosynthesize glycogen. However, we found no significant differences in the glycogen levels between samples from the TN1- and Mudgo-BPHs (Fig. 3D).

### Identification and functional annotations of the differentially expressed genes (DEGs)

To generate the transcriptional information during the compatible and incompatible rice-BPH interactions, transcriptomes of Mudgo-BPH and TN1-BPH fed on the Mudgo rice were sequenced by RNA-Seq. After filtering low-quality reads, the average numbers of clean reads were 46.44 (54.13, 38.75) and 48.13 (55.13, 41.13) million for the sequencing libraries of Mudgo-BPH and TN1-BPH, respectively. The whole sequence reads were deposited in the NCBI Sequence Read Archive (BioProject: PRJNA533704). Approximately 80.86% of the clean reads were mapped to the BPH genome assembly for the four sequencing libraries. From the mapped clean reads, 16,482 genes were expressed in more than one biological replicate (FPKM > 0) and were comparatively analyzed in the two groups of BPH. In total, 538 genes were found to exhibit significant differential expressions in between Mudgo-BPH and TN1-BPH (adjusted *p*-value < 0.05) (Supplementary Table S1, Fig. 3). Out of these, several genes involved in the key BPH physiological processes, including the formation of cuticle (NLU012128.1, NLU008431.1), detoxification (NLU006811.1, NLU025857.1), metabolite transport (NLU004874.1, NLU018331.3), digestion (NLU021394.1, NLU007691.1) and RNA processing (NLU014287.2, NLU015867.1) were significantly down-regulated in the TN1-BPH as compared to the Mudgo-BPH (*P*_adj_ < 0.05). In addition, genes encoding insulin receptor 2 (InR2) (NLU008487.1) and insulin-like peptide 1 (ILP1) (NLU003562.3), and tumor-necrosis factor receptor-associated factor 5 (TNF5) (NLU005471.1) having crucial roles in the insect insulin signaling were found to be significantly upregulated in TN1-BPH as compared to the Mudgo-BPH. Besides, genes involved in the lipid or fatty acid metabolisms (NLU018257.1, NLU015779.1) in BPH were also significantly downregulated in the TN1-BPH fed on the Mudgo rice as compared to the Mudgo-BPH. Important genes like *serpin* (NLU002506.1) encoding the protease inhibitor from the serine protease inhibitors class were also found to be significantly downregulated in the TN1-BPH than in the Mudgo-BPH. On the other hand, genes involved in processes like amino acid response (NLU023320.1, NLU015341.1), chaperons (NLU005951.1, NLU022358.1), histone protein modifications (NLU023233.1, NLU024917.1), and locomotive-related proteins (NLU002237.1, NLU011145.1) were found to exhibit significant upregulation in their transcript levels in the TN1-BPH as compared to the Mudgo-BPH.

### GO and KEGG enrichment analysis of the DEGs

**Figure 4:**
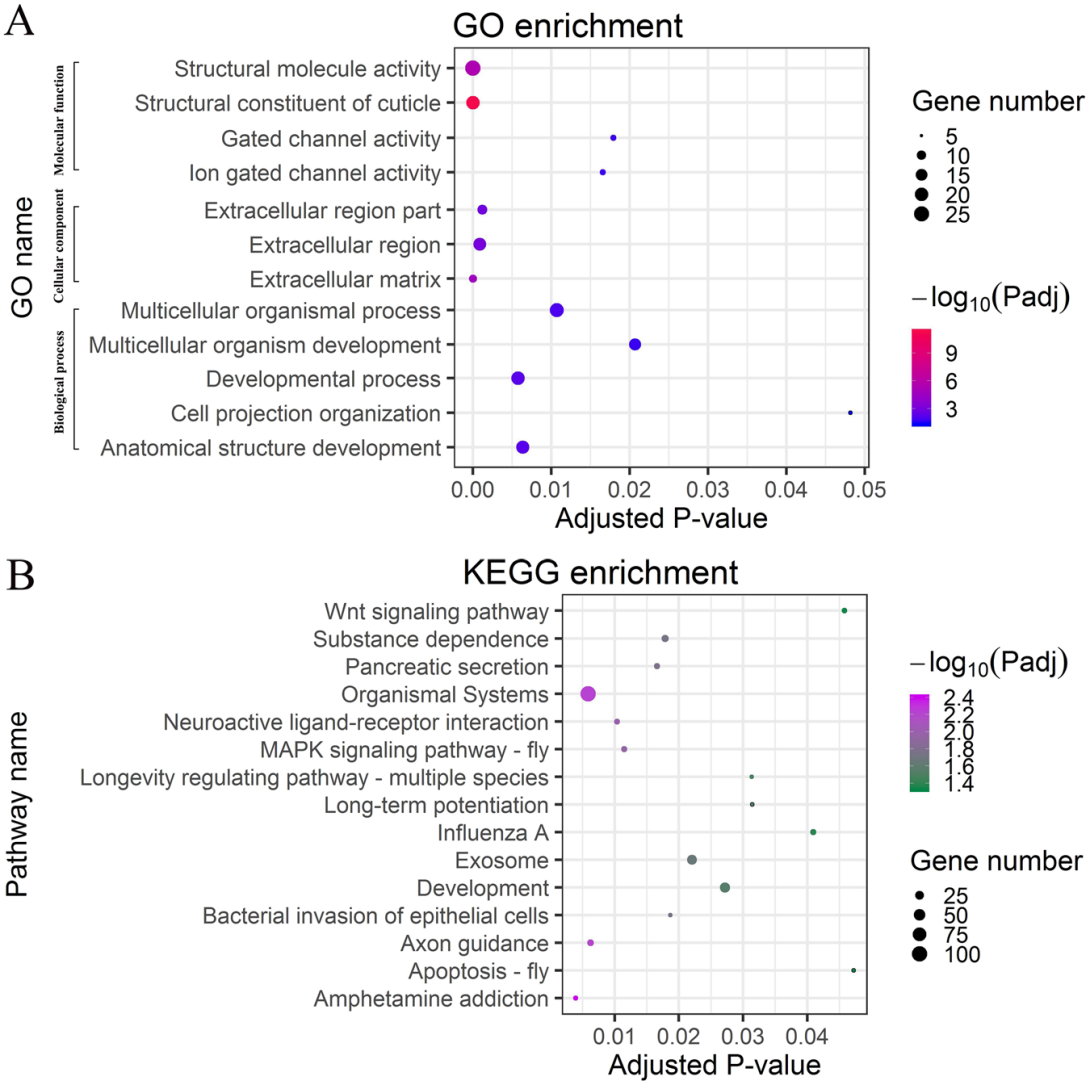
GO functional (**A**) and KEGG pathway (**B**) enrichment of differentially expressed genes. Dots represent GO term or KEGG pathway enrichment where the color coding indicates the magnitude of *P*-values. The sizes of the dots represent the count of each row (GO or KEGG category).

To deduce the functional annotations of the identified DEGs in TN1-BPH, the GO enrichment studies were performed for all the DEGs. The results revealed that 41 of the 300 significantly downregulated genes in the TN1-BPH were enriched for twelve GO categories (*P*_adj_ < 0.05) that grouped into three ontologies (biological process, molecular function, and cellular component) (Fig. 4A). Out of those, structural molecule activity (GO:0005198), extracellular region (GO:0005576), and developmental process (GO:0032502) had 19, 9, and 12 genes, respectively (Supplementary Table S2). Similarly, out of 238 upregulated genes in the TN1-BPH, 50 genes were found to be functionally enriched with different GO terms and grouped into the three different ontologies.

The KEGG analysis of the identified DEGs in the TN1-BPH was performed to deduce the pathway enrichments for the DEGs. The results exhibited that in three (long-term potentiation, neuro-active ligand-receptor interaction, amphetamine addiction, and organismal systems) of the enriched pathways, one down-regulated gene (NLU028910.1) was found to be 100% identical to one of the members of glutamate receptors in BPH (XP_022194510.1). Furthermore, three genes of interest in the BPH (vitellogenin-1-like, *CYP6AY1*, and sugar transporter) were also found in the organismal systems pathway. Moreover, we found that six genes were enriched in six pathways, including exosome, organismal systems, mitogen-activated protein kinase (MAPK) signaling pathway, amphetamine addiction, substance dependence, and long-term potentiation (Fig. 3B, Table S3). These genes encode two types of enzymes those are vital for the post-translational modifications: 1) the protein kinases (PKs, which are encoded by NLU005508.1, NLU018444.1, NLU003761.1, and NLU014735.1), responsible for the protein phosphorylations, 2) the protein phosphatases (PPs, which are encoded by NLU002162.1, NLU012434.1), responsible for the dephosphorylations.

### Expression validation and knockdown of the selected DEGs

**Figure 5:**
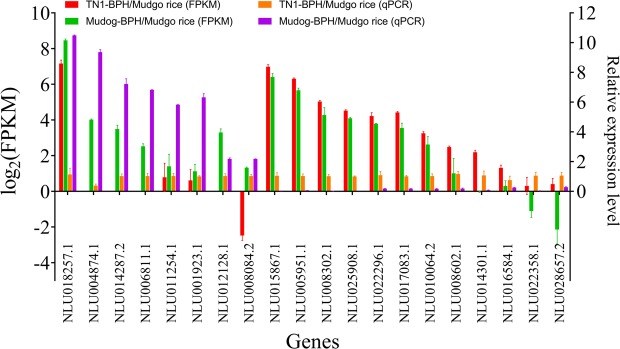
The relative expression level change of 12 selected DEGs between TN1-BPH and Mudgo-BPH as analyzed by quantitative real-time PCR. Differently expressed genes selected from the transcriptome for FPKM analysis and in real-time PCR for 2^−ΔΔCT^ analysis. The FPKM values were shown as log_2_(FPKM).

The expressions of 20 randomly selected DEGs were determined using RT-qPCR in the TN1-BPH and Mudgo-BPH to compare the transcript abundances with the RNA-seq data. Although, the results revealed marginal differences in the fold change values in between the RT-qPCR and the RNA-seq data, the significance of the relative expression of the DEGs between TN1- and Mudgo-BPH were in concordant with the RNA-seq results, indicating the reliability of the gene expressions (Fig. 5).

**Figure 6:**
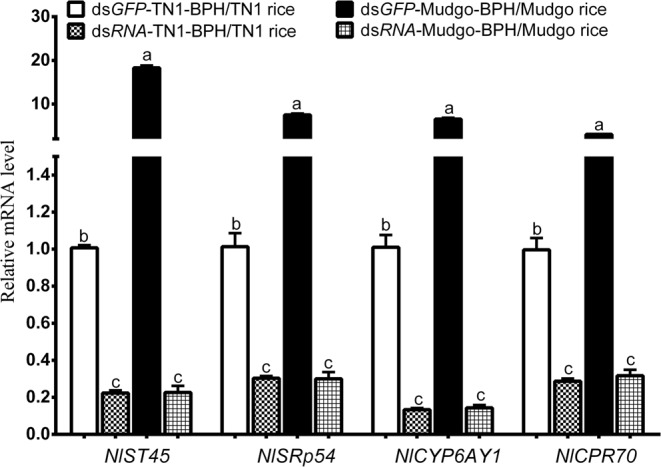
Effect of dsRNA (ds*NlST45*, ds*NlSRp54*, ds*NlCYP6AY1*, and ds*NlCPR70*) on the expression levels of their coresponding targets. The bars represent 2^−ΔΔCT^ values ( ± SE) normalized to the geometrical mean of the housekeeping gene expression. SE was determined from three independent biological replicates, each with three technical replications. Data are analyzed by one-way ANOVA followed by Tukey’s HSD post-hoc test. Different letters indicate a significant difference at *P* value < 0.05.

**Figure 7:**
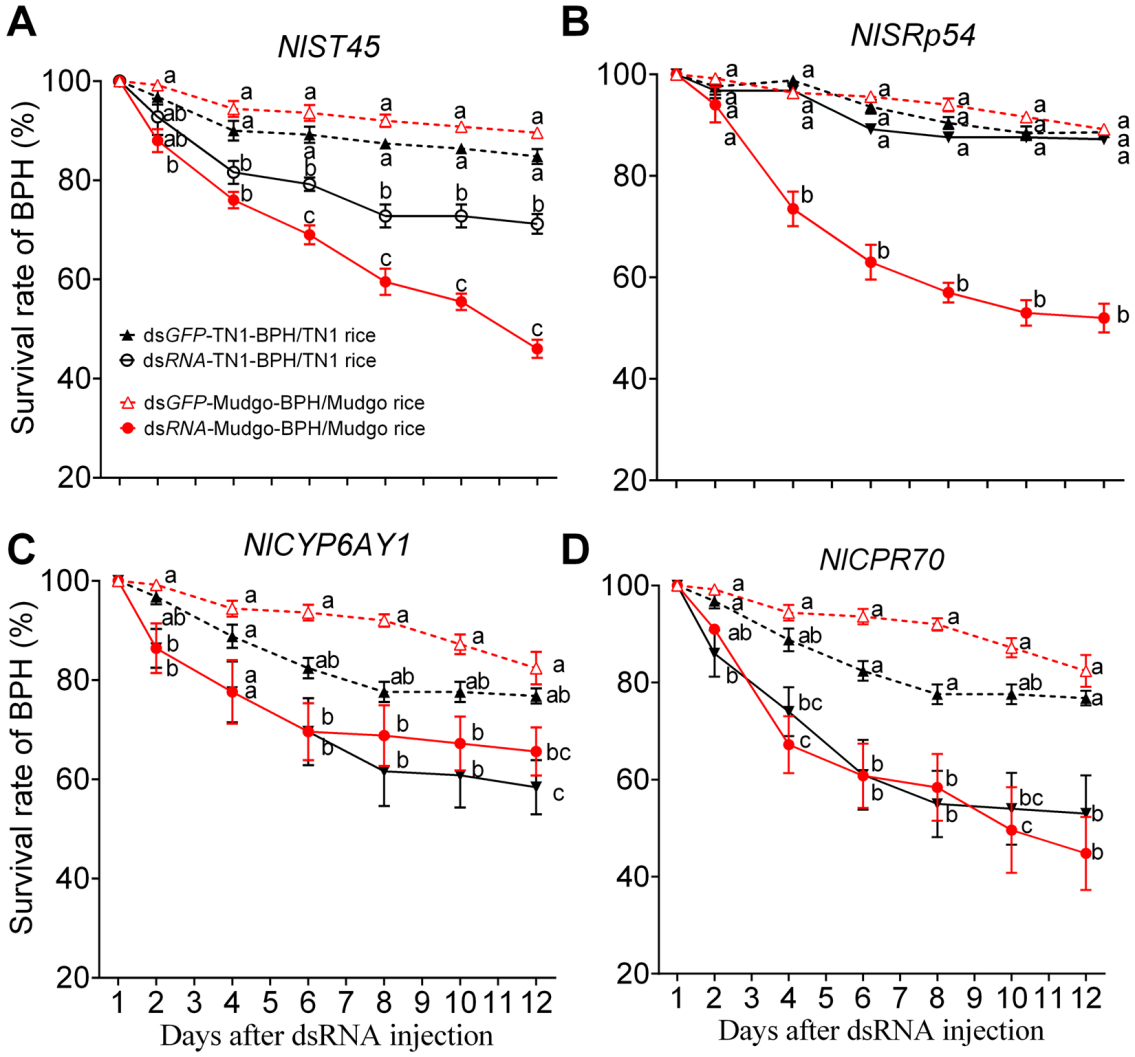
The survival rates of BPHs on rice. The survival rate of the RNAi treatment (the dsRNA-treated TN1-BPHs were feeding on TN1 rice, and the dsRNA-treated Mudgo-BPHs were feeding on Mudgo rice. The BPHs, injected with ds*GFP* were taken as control. Data are denoted as mean ± SE and analyzed by two-way ANOVA followed by Tukey’s HSD post-hoc test. Different letters indicate significant differences at *P* value < 0.05.

According to the functional annotations of the DEGs, 4 genes of interest, including NLU004874.1 (encoding NlST45, a sugar transporter), NLU014287.2 (encoding SRp54, a serine and arginine-rich protein), NLU006811.1 (encoding CYP6AY1, a cytochrome P450 protein), and NLU012128.1 (encoding CPR70, a cuticle protein) with significant lower transcript abundance in TN1-BPH (FPKM = 0), but higher in Mudgo-BPH (FPKM > 6) were selected to determine their functions by RNAi method. The dsRNAs for each of the genes had been successfully synthesized and were injected into the fourth instar nymphs of TN1-BPH or Mudgo-BPH. BPH nymphs injected with *dsGFP* served as controls to evaluate the efficacy of the dsRNAs in target gene silencing and the BPH bioassays results. Subsequently, the qRT-PCR assays confirmed that all target genes were efficiently suppressed in the BPHs (Fig. 6). The BPH assay results revealed that the DEGs are essential for the BPH survival for both TN1- and Mudgo-BPH populations on the Mudgo rice (Fig. 7). The administration of *dsNlst45* into the TN1-BPH nymphs resulted in the significant (*P* < 0.05) reduced BPH survival rates after four days of the dsRNA injection (Fig. 7A). Likewise, injection of *dsNlst45* into the Mudgo-BPH nymphs caused significant (*P* < 0.05) decreased BPH survival rates after two days of the dsRNA injection. While, the BPH survival rates were ultimately brought down to below 80% in the TN1-BPH, the Mudgo-BPH survival rates were further declined to below 50% at 12 days after the *dsNlst45* injection. Knockdown of another DEG, *NlSRp45* by using the *dsNlSRp45* resulted in the significant (*P* < 0.05) Mudgo-BPH survival rate reduction after 4 days of the dsRNA administration (Fig. 7B). However, the knockdown of *NlSRp45* in the TN1-BPH didn’t cause any significant loss in the BPH survival rates as compared to the *dsGFP* injected controls. Similarly, silencing of *NlCYP6AY1* resulted in the significant reduction in the Mudgo-BPH survival rates after two days of the dsRNA injection (Fig. 7C), ultimately bringing down the BPH survival rates to just above 70% at 12 days of the post-dsRNA treatment. However, the silencing of *NlCYP6AY1* in TN1-BPH exhibited a delayed change in the BPH survival rates, significantly reducing it at 12 days of the post-dsRNA treatment. Lastly, the knockdown of *NlCPR70* decreased the TN1-BPH and Mugdo-BPH survival rates significantly at 2 and 4 days of the post-dsRNA treatment, respectively, decreasing the BPH survival rates down to <60% at 12 days of the post-dsRNA treatment (Fig. 7D). The silencing of *Nlst45* or *NlSRp45* or *NlCPR70* caused significant reductions (<60%) in the Mudgo-BPH survival rates as compared to that of the control group (*dsGFP* injected, >80%), which suggested their indispensable roles in the nymph/adult development of BPHs.

## Discussion

In the present study, the comparative evaluation of the performance and transcriptomic dynamics of two different *N. lugens* populations (the avirulent TN1-BPH and the virulent Mudgo-BPH) infested on the Mudgo rice have been realized. Firstly, the feeding behaviors as analyzed by the EPG records revealed that the TN1-BPH had a longer NP and shorter (discontinuous) phloem feeding (N4) phase, whereas the Mudgo-BPH had a longer (continuous) N4 and a shorter NP phase, while feeding on the resistant Mudgo rice. Also, the amount of honeydew measured, indicated that the TN1-BPH didn’t have a continuous phloem ingestion as compared to the Mudgo-BPH. Longer N4 and shorter NP phases during the compatible interactions between BPH and the susceptible rice genotypes, including TN1, Nipponbare, IR694, and Azuceana, whereas longer NP and shorter N4 phases during the incompatible interactions between BPH and resistant rice genotypes, including Rathu Heenathi, Babawee, and IR64 have been reported^22^. Similar patterns of the feeding behaviors were also reported during the black currant-lettuce aphids and lettuce genotypes, where the incompatible interactions had longer NP and shorter phloem ingestion (E2) phase and vice versa for the compatible interactions^30^. Moreover, this differential feeding behavior might have significant effects on the overall fitness of the TN1-BPH, as the avirulent TN1-BPH exhibited lower honeydew productions and a negative weight gain on the Mudgo rice.

Secondly, the reduced ingestion of phloem sap during the incompatible rice-BPH interactions might affect the BPH physiology, especially the sugar metabolisms. In this study, the glucose and trehalose assays revealed that feeding on the resistant Mudgo rice caused a significant reduction in the glucose and trehalose levels in the TN1-BPH. Sucrose is the main sugar present in the phloem sap, which is catabolized to produce glucose and fructose in the insect body^31^. Further, trehalose being the major sugar of the insect haemolymph is synthesized from glucose in an energy consuming process^32^. Feeding on a resistant rice (near isogenic line carrying *Bph15*) for 12 hours had resulted in the decreased levels of glucose and TCA cycle intermediates in the BPH^21^. However, shifting the BPH from the resistant rice to the susceptible TN1 rice resulted in the metabolite restorations, including a rise in the levels of trehalose after 24 hours of feeding^21^. In this study, the discontinuous phloem ingestion by the TN1-BPH resulted in the starvation of sucrose in the BPH, leading to the reduced glucose and trehalose levels. As a feedback response to the lower blood glucose levels, the BPH might have urged to feed more on the Mudgo rice, which can further be supported by the prolonged NP phase trying to accumulate nutrients.

Thirdly, the transcriptome analysis of TN1- and Mudgo-BPH revealed the differential transcript abundance in between the two BPH populations upon feeding on the Mudgo rice. A previous study on the salivary gland transcriptomes of TN1- and Mudgo-BPH maintained on TN1 and Mudgo rice, respectively, reported 3757 numbers of differential transcripts involved in the vital processes, including carbohydrate metabolism, digestion and absorption, and salivary secretion^23^. Similarly, 27 and 7 genes were found to get upregulated and downregulated, respectively in the BPH salivary gland, when the BPH feeding on a resistant rice (B5) was shifted onto a susceptible rice (TN1)^24^. In this study, the functional annotations of the DEGs revealed that they might be involved in crucial physiological processes in BPH, including cuticle formation, sugar transport, detoxification, development process, protease inhibition, lipid metabolism, and insulin signaling. For instance, the feedings on Mudgo rice resulted in the downregulation of two insulin signaling (ILS) players, insulin receptor 2 (InR) (NLU017789.1) and tumor-necrosis factor receptor-associated factor 5 (TNF5) (NLU005471.1) in the TN1-BPH. The TNFs in *Drosophila* have been reported to be involved in the JNK-dependent inhibition of insulin production^33^, whereas the InRs act as the binding protein for the insulin peptides (ILPs) during the ILS^34^. Additionally, lower levels of InRs are associated with the insect foraging and food search, as the constitutive expression of *InR*s in the *Drosophila* larva reduced its foraging movements^35^. Overexpression of *ILP*s in *Drosophila* larvae increased the food intake and enable them to switch onto the non-preferred food^35^. Furthermore, the ILS can enhance the food foraging by inducing the expression of olfactory senses of the starving insects^36^. In this study, the downregulation of *InR2* and *TNF5*, while the up regulation of *ILP1* (NLU003562.3) suggests the role of ILS in regulating the feeding activity of the TN1-BPH on the non-preferred phloem sap of the resistant Mudgo rice, and in the wing morph switch of BPHs response to two most relevant environmental cues, host quality and population density^37^. Moreover, the longer NP phase and upregulated expressions of the chemosensory/olfactory genes, including the odorant-binding protein 8 (*OBP8*) (NLU009751.1), *OBP9* (NLU014301.1), and chemosensory protein (*CSP9*) (NLU026323.1) further supports this hypothesis. Within population or species (named ‘taxa’), the substantial transcription dynamics can be observed. In addition, differences in the gene expressions, including both variable and heritable can be affected by processes like natural selection and neutral drift. It has been reported that extensive diverse gene expressions can be observed within the widely separated taxa, if the genetic variation is put through neutral drift^38^. Conversely, through natural selection, the divergence among taxa is modulated by the local ecological state. Noteworthy to mention that gene expression can also be affected by the epigenetic factors, which might have been experienced by the BPH populations while feeding on the Mudgo rice.

Lastly, the independent gene silencing experiments via RNAi of the selected DEGs involved in the vital BPH physiological processes, including sugar transport (NLU004874.1), mRNA processing (NLU014287.2), detoxification (NLU006811.1), and cuticle formation (NLU012128.1) revealed their functional roles in BPH survival and development. Knocking down a serine/arginine (SR)-rich splicing factor encoding gene *NlSrp54* caused significant decrease in the Mudgo-BPH survival rates. The SR family splicing factors, such as Srp54 are involved in the mRNA alternative splicing events of many organisms, including *Saccharomyces cerevisiae*, *D. melanogaster*, *Aedes aegypti*, *Oryza sativa*, and humans^39,40^. Further, the alternative splicing events have been associated with a gain or loss of virulence in several organisms, including fungi^41^ and protozoans^42^. Thus, silencing *NlSrp54* in the virulent Mugdo-BPH population might have impaired the release of a necessary transcript variant, which might have assisted the survival of Mudgo-BPH on the resistant Mugdo rice. In addition, the knockdown of *Nlst45*, encoding a sugar transporter, resulted in the significant reduced survival rates of both TN1- and Mudgo-BPH. The necessary monosaccharides, including glucose and fructose are transferred across the BPH body by different sugar transporters^31^. Thus, silencing *Nlst45* might have affected the sugar transports in BPH, eventually affecting the sugar metabolism, energy production, and overall fitness. Similarly, silencing *NlCYP6AY1*, encoding a cytochrome P450, reduced the BPH survival rates of the TN1- and Mudgo-BPH <70%. The insect cytochrome P450 family proteins have been reported to be involved in the detoxification of xenobiotics, including insecticides and toxic plant secondary metabolites^43,44^. Therefore, a functional *NlCYP6AY1* in the Mudgo-BPH might have aided its survival on the resistant Mudgo rice, possibly by detoxifying the plant-produced toxic secondary metabolites. However, knocking down *NlCYP6AY1* might have hindered the plant allelochemical detoxifications, resulting in the decreased BPH survival rates. Additionally, the silencing of a BPH structural protein *NlCPR70*, encoding a cuticle protein, brought the BPH survival rates of both the populations down to <50%. Cuticle is a major structural protein in insects having crucial roles in insect development and resistance^45^. Reasonably, the knockdown of *NlCPR70*, the protein involved in the exoskeleton formation, in the BPH populations might have affected a plethora of physiological processes, including mechanical support, locomotion, sensory perception, desiccation, and resistance response^45^, resulting in the BPH fatality.

## Conclusion

In conclusion, the current study revealed the differential feeding behavior, biochemical, and transcriptomic dynamics of the TN1- and Mudgo-BPH, having different virulence levels, during their infestation on the resistant Mugdo rice. The EPG data records showed that the TN1-BPH feeding behaviors are associated with a typical incompatible rice-BPH interaction having longer NP and shorter N4 phase, whereas the Mudgo-BPH feeding was associated with a typical compatible rice-BPH interaction having shorter NP and longer N4 phase. Glucose and trahalose biochemical assays confirmed that shifting the TN1-BPH from the susceptible TN1 rice onto the resistant Mudgo rice caused significant decrease in the glucose and trehalose levels, respectively. The transcriptome sequencing and the functional annotations by GO and KEGG analysis revealed several DEGs in the TN1-BPH to be involved in many crucial BPH physiological processes, including development, digestion, transport, sugar and lipid metabolism, detoxification, insulin signaling, and locomotion. Gene silencing via RNAi of the selected DEGs revealed their indispensable roles in BPH development and survival. Moreover, the current work adds new insights into the rice-BPH interactions and depicts the transcriptomic and physiological differences in between two BPH populations of different virulence levels with reference to their infestation on a resistant rice variety. Overall, the results from this study will be helpful for the future researches focusing on the virulence shifts of BPHs and the rice resistance breakdown.

## Materials and Methods

### Ethics statement

All animal work has been conducted according to the relevant national and international guidelines.

### Insects and plant materials

Two BPH populations (an avirulent TN1-BPH that is incapable to break down resistance of the rice varieties containing *Bph* genes and a virulent Mudgo-BPH having the capacity to break down *Bph1*-mediated resistance) were reared on rice varieties TN1 (susceptible variety) and Mudgo (with *Bph1* gene), respectively, for more than 170 generations in China National Rice Research Institute (CNRRI) at 28 ± 2 °C and 80 ± 5% relative humidity under a 14/10 h light/dark photoperiod. The newly emerged brachypterous females were collected from TN1 rice and Mudgo rice and then were separately reared on Mudgo for two days. After two days of infestation on Mudgo rice, the BPH individuals from both populations i.e. TN1-BPH (named as TN1-BPH/Mudgo) and Mudgo-BPH (named as Mudgo-BPH/Mudgo) were collected for bioassays, trehalose and glycogen assays, and total RNA isolation.

### BPH bioassays and electrical penetration graph (EPG) recording

For bioassays, a previously reported procedure was used to analyze the BPH growth rates^18^. Briefly, after weighing the newly emerged brachypterous female and Parafilm sachets (cm × 2.5 cm), the individual was placed into the sachet, which was then attached to the Mudgo rice plant (45 days after sowing). After 48 h, the insect and sachet were reweighed, and the changes in weight of the BPH and sachet were defined as BPH weight gain and honeydew weight, respectively. The experiment was repeated 50 times per group, and the experiments were conducted five times.

The feeding behavior of BPH was recorded for 6 h on a CR-8 direct current (DC) EPG amplifier (CNRRI, Hangzhou, China)^46^. One end of the gold wire (20 μm in diameter and 4 cm in length) was attached to the dorsal thorax of the BPH with a water-soluble conductive silver glue and the other end of the wire was connected to the amplifier. The signals recorded were analyzed using insect feeding behavior signal sampling and processing system v1.0 (CNRRI). The output signals from EPG recordings were classified into five different waveforms according to the relative voltage level^22^, including PP (N1 + N2 + N3) for the “pathway” phase (including penetration initiation, salivation and stylet movement, and extracellular activity near the phloem), N4 for continuous phloem sap ingestion (N4-a for intracellular activity in the phloem, N4-b for phloem sap ingestion), N5 for the xylem ingestion, NP, and unclear waveform type (N6 for derailed stylet mechanics, N7 for cell penetration). All experiments were carried out at 26 ± 2 °C and 65 ± 5% relative humidity under continuous light conditions. Fifteen independent replications were recorded and the experiments were repeated three times.

### Glycogen, glucose and trehalose assays

The content assays of glycogen, glucose, and trehalose were performed by following the methods described^47^. Briefly, samples were homogenized in 1 ml pre-cooled phosphate buffered solution (0.02 M, pH 6.0). The solution was then centrifuged at 1000 × g for 20 min at 4 °C. The supernatant was divided into two portions with an identical weight (350 μL). The first portion was further divided into two groups to measure the glycogen and trehalose content, respectively. The glycogen or trehalose content was determined using the glycogen assay kit or the trehalose assay kit (Comin, Suzhou, China) respectively, following the manufacturer’s instructions. The second portion was centrifuged at 20800 × g for 60 min at 4 °C. The supernatant was used to measure the glucose content which was determined using a Glucose (GO) Assay Kit (Sigma-Aldrich, Shanghai, China), following the manufacturer’s instructions. The contents were given as micrograms per milligram fresh body. Controls were prepared in the absence of the enzyme, and the amount of glycogen, glucose, or trehalose were calculated by excluding the endogenous glucose. The experiment was replicated six times.

### RNA-Seq data analysis

Total RNA from the TN1-BPH (forty-one individuals) and Mudgo-BPH (forty-five individuals) fed on the Mudgo rice for two days was isolated using the TRIzol Reagent (Invitrogen, Carlsbad, CA, USA) by following the manufacturer’s instructions. The concentration and quality were examined by a NanoDrop 1000 spectrophotometer (Thermo Fisher Scientific, Rockford, IL, United States) and 1% agrose gel electrophoresis. The cDNA libraries were constructed from the isolated total RNA and sequenced on an Illumina HiSeq 2000 sequencing platform at BGI-Shenzhen (Shenzhen, China). The high-quality clean reads were generated by trimming the raw reads with adaptors, reads where the number of unknown bases was more than 10%, and low-quality reads (the percentage of the low-quality bases with which value ≤5 was more than 50% in one read). The clean reads were mapped to the *Nilarpavata lugens* reference genome (GCA_000757685.1) using Hisat2 v2.1.0^48^. Then, the transcript level raw read count matrix and FPKM (Fragments Per Kilobase of transcript per Million fragments mapped) were extracted directly from the files generated by StringTie v1.3.5^49^ using a Python script (http://ccb.jhu.edu/software/stringtie/dl/prepDE.py). The count matrix was further processed by DESeq2^50^ to estimate the differential expressed genes (DEGs). The adjusted *P* values (*P*_adj_ ≤ 0.05) and an absolute value of log_2_Ratio ≥ 1 were used as cutoffs for the statistical significance. The experiments were repeated two times.

Gene ontology (GO) categories were assigned to all the genes via a BLASTX hit using the Blast2GO software^51^. The DEGs were first mapped to the GO terms using a standard database (http://www.geneontology.org/); the gene numbers for each term were calculated, and the GO terms significantly enriched in the DEGs as compared to the background genome were determined with a hypergeometric test. All calculated *P* values were then subjected to Bonferroni Correction, using a corrected *P* ≤ 0.05 as the threshold. GO terms that fulfilled this criterion were defined as significantly enriched in the DEGs. On the basis of the Kyoto Encyclopedia of Gene and Genome (KEGG), the pathway enrichments were then analyzed using the same method. The candidate DEGs were then randomly selected to confirm this analysis by quantitative real-time polymerase chain reaction (qRT-PCR).

### Expression analysis by qRT-PCR

The total RNA (~1 μg) was reverse transcribed to cDNA by using the ReverTra Ace qPCR RT Master Mix with gDNA Remover Kit (Toyobo. Co. Ltd., Osaka, Japan) following the manufacturer’s manual. The qRT-PCR experiments were carried out using the SYBR Green Realtime PCR Master Mix (Toyobo) according to the manufacturer’s instructions, on an ABI 7500 real time PCR System (Applied Biosystems, Foster City, USA). The transcript abundance was estimated using *tub* (ACN79512.1) and *rps15* (ACN79501.1) (primers are listed in Supplementary Table S2) as internal reference genes^52^. The data were analyzed according to the 2^−ΔΔCT^ method^53^ using the geometric mean of *tub* and *rps15* for normalization^54,55^. An RT negative control (without reverse transcriptase) and a non-template negative control (NTC) were included for each primer set to confirm the absence of genomic DNA and to check for primer-dimer or contamination in the reactions, respectively. Each sample contained three biological replicates and each biological replicate had the technical triplicates. All methods and data collections were confirmed to follow the MIQE (Minimum Information for Publication of Quantitative Real Time PCR Experiments) guidelines^56^.

### Synthesis of dsRNAs and RNAi bioassay

The dsDNA fragment was PCR amplified by using specific primers (Supplementary Table S2) conjugated with the T7 RNA polymerase promoter (5′-taatacgactcactataggg-3′). The PCR products were gel purified and used as templates to synthesize dsRNA using MEGAscript T7 High Yield Transcription Kit (Ambion, Austin, USA). The dsRNA of the enhanced green fluorescent protein (ds*EGFP*) was used as a negative control for any nonspecific effects of dsRNA.

RNAi bioassays were performed by injections as previously reported^57^. Briefly, 200 ng (0.05 μl) dsRNA (concentration estimated as 4 mg/ml) or the same amount of ds*GFP* (negative control) was injected into the newly emerged fourth-instar nymphs of TN1-BPH and Mudgo-BPH^37^, and reared on TN1 rice and Mudgo rice. A total of 140 nymphs (7 replicates, 20 individuals in each replicate) for each treatment was used for each dsRNA injection. Three replicates were used for survival evaluation, three replicates for qRT-PCR, and one replicate was kept as a backup replication. To confirm RNAi, at 4–6 days after injection, total RNA was isolated from the individuals of the qRT-PCR replicates to check the transcript levels of the target genes. Three technical replicates for each biological replicate were performed during qRT-PCR.

### Data analyses

Data analysis was carried out using the Data Processing System software^58^. Data are reported as mean ± SE. The student’s *t*-test was applied to the comparisons of two samples and one- or two-way analysis of variance (ANOVA) followed by Tukey’s HSD post hoc test was applied for three or more samples. The statistical significance level was set to *P*-values < 0.05 or 0.01.

## Supplementary information

Supplementary Table S2

Supplementary Table S1
