# Supplementary material for: Phenotypic and transcriptomic responses of two Nilaparvata lugens populations to the Mudgo rice containing Bph1
Source: Sci Rep. 2019 Oct 1;9:14049. doi: 10.1038/s41598-019-50632-z (PMC6773769; doi:10.1038/s41598-019-50632-z)
Supplement: Supplementary file 1 — Supplementary Table S2 [file 41598_2019_50632_MOESM1_ESM.doc]

**Supporting Information**

**Phenotypic and transcriptomic responses of two *Nilaparvata* *lugens* populations to the Mudgo rice containing *Bph1***

Pin-Jun Wan^#^*, Ruo-Nan Zhou^#^, Satyabrata Nanda, Jia-Chun He, San-Yue Yuan, Wei-Xia Wang, Feng-Xiang Lai, Qiang Fu*

State Key Laboratory of Rice Biology, China National Rice Research Institute, Hangzhou 310006, China

#These authors contributed equally to this work.

*Correspondence and requests for materials should be addressed to P.-J.W. (email: wanpinjun@caas.cn) or Q. F. (email: fuqiang@ caas.cn)

**Supplementary Table S1. List of differentially expressed genes in the TN1-BPHs and Mudgo-BPHs feeding on Mudgo rice.**

| **Gene ID or name** | **Amplicon Size (bp)** | **Forward sequence (5′-3′)** | **Reverse sequence (5′-3′)** |
| --- | --- | --- | --- |
| **qPCR** |  |  |  |
| NLU014287.2 | 123 | GGCTGGCAAAGTGATACAGG | CTCTGTGCTTCCTTGACACG |
| NLU011254.1 | 141 | GCCGCTTCGACAAGAGAATG | TGTGACGCATCAGGTTGTTC |
| NLU008602.1 | 294 | GGAGGCTCTCTCATCTCTCG | ACCTTCCACACCCTCAAACA |
| NLU001923.1 | 105 | GGTGCCTGAAAGTCGTGTAT | TTGGCAGGCAAAGCTATCAA |
| NLU006811.1 | 183 | TGTTTCACTTCTTGAGACTCCGC | TTCTAAATCATTAGCCTCCGTTC |
| NLU011510.2 | 275 | TTGGGACCGCTATGCTGCTG | CGTTACTGGTCGCCGTGTCA |
| NLU028657.2 | 125 | CGCACTCGTGTCACCCGTTA | TGTGAACCCGTCCGAATCGC |
| NLU010064.2 | 144 | GCATTCTGGGCCCACAGGTT | CTGAGTCGTCGTCGCTGTCC |
| NLU012128.1 | 142 | CGTTAAGGTACTGTCAATGT | GGTAGGCGAACTGGTATT |
| NLU005951.1 | 167 | CGACGCATCGGTCATCGTCA | TGGTCAACAGCGACGCAACT |
| NLU022358.1 | 192 | CCGAGGACAGCCGAATACCC | AGCGGTGACAGAAGTGTCGAG |
| NLU004874.1 | 115 | TGATGACAGCCCTGGTCCGA | GAGGTGGCCATAGCCGTTCC |
| NLU014301.1 | 155 | GGCAAGCTGAACGTGGAAGC | AGCTCGTGGGATCCACTGGT |
| NLU016584.1 | 94 | TGCGCAAACGCATCAAAGCC | GCCAGTTTGACGGCCTCCTG |
| NLU022296.1 | 146 | GCGCACTTCGCCATGAACTG | CCAGCCACTATGCCGACGAG |
| NLU008302.1 | 104 | AGAGGGTCGTGCTGTCAGGT | GACCGGCTTGAACCAAGCGA |
| NLU017083.1 | 146 | CCAGATTGTGCCGGAACGGA | GGCGCCATGCCTTGACTGTA |
| NLU004886.1 | 107 | ATGAGGCGTGCATTGAGCGA | GGGAATGCCGTGGCAGATGA |
| NLU014460.1 | 117 | CGAGTTCGGAGTGCTGCTGT | GCCGGAGTGGCGAATAGGAG |
| NLU025908.1 | 101 | GCGCGGGCTGGAGAAATCA | AAATGGCGAATGGCGGCAAC |
| NLU018257.1 | 191 | CAGGTTTGGACCCGGCTCAG | TCCGGGCTGATCGTAACCAC |
| NLU023215.1 | 215 | GTGCGGCGACAAACAACTCC | CACCGACCAATCGGCATCCA |
| NLU015867.1 | 229 | ACTTGGGCGAACGCATCACA | GTGACACTGGCCGACTGCTT |
| NLU004890.1 | 112 | GTGGCCAGCATTGTCACCCT | ACCTTCCAGGCCTTCGCAAC |
| NLU008084.2 | 93 | TGCCAAGCCCTGAGTCACTG | GCTAGTCCAGCCCAGCTTGT |
| *RPS15* | 150 | TAAAAATGGCAGACGAAGAGCCCAA | TTCCACGGTTGAAACGTCTGCG |
| *Tub* | 174 | ACTCGTTCGGAGGAGGCACC | GTTCCAGGGTGGTGTGGGTGGT |
| **dsRNA synthesis** |  |  |  |
| NLU014287.2 | 634 | CACTTTTTGGATTCATAGGG | TGTATCACTTTGCCAGCC |
| NLU006811.1 | 305 | TTGGAAGCAACGCAGTGT | TTCGCCATTTTTCGCCCT |
| NLU012128.1 | 281 | CATACGCTGGAGTGGTTC | CAGTAAGTGGCAGTTGTCT |
| NLU004874.1 | 389 | CACATGGAGCAGATGAGGAT | ATGGGGGTGGCTATTGAA |
| *GFP* | 460 | CACAAGTTCAGCGTGTCCG | GTTCACCTTGATGCCGTTCT |
